# Adjuvant radiotherapy for WHO grade II meningiomas: the unanswered question

**DOI:** 10.3389/fonc.2026.1815992

**Published:** 2026-06-24

**Authors:** Alice Giotta Lucifero, Ossama Al-Mefty

**Affiliations:** 1 Department of Neurosurgery, Mass General Brigham, Harvard Medical School, Boston, MA, United States; 2 Department of Brain and Behavioral Sciences, University of Pavia, Pavia, Italy

**Keywords:** adjuvant radiotherapy, atypical meningioma, meningioma recurrence, radiosurgery, WHO grade II meningiomas

## Abstract

**Background:**

WHO grade II meningiomas have a known propensity for recurrence and aggressive clinical behavior. The role of adjuvant radiotherapy after surgical resection remains the subject of ongoing debate, with existing studies showing conflicting conclusions. This study analyzes the impact of adjuvant radiotherapy on progression-free survival (PFS) and cause-specific survival (CSS), focusing on the extent of resection and proliferative index.

**Methods:**

We retrospectively analyzed 318 patients with WHO grade II meningiomas treated from 1990 to 2023. All patients underwent surgical resection; 104 received adjuvant radiation therapy. We assessed the survival outcomes using Kaplan–Meier curves and Cox proportional hazards models. Subgroup analyses were performed based on the extent of resection (gross total [GTR] vs. subtotal [STR]) and Ki-67 index (<10% vs. ≥10%). The data were fit to competing risk models by estimating cause-specific hazard functions.

**Results:**

At a median follow-up of 5.1 years, adjuvant radiotherapy was associated with significantly reduced PFS after both GTR and STR (p<0.0001) and in both Ki-67 index groups. CSS was also less following radiotherapy after GTR (p=0.0237). Stereotactic fractionated conformal radiotherapy and radiosurgery were independent predictors of recurrence and death. Competing risk models revealed increased hazards of recurrence and mortality across all irradiation modalities.

**Conclusions:**

Adjuvant radiotherapy is significantly associated with decreased PFS and CSS, without statistical significance between the GTR and STR groups. In the GTR group, radiation was associated with worse survival outcomes compared to surgery alone. These findings contradict some current treatment paradigms and highlight the urgent need for personalized approaches and further prospective studies with precise parameters to provide a definitive answer.

## 1 Introduction

Meningiomas account for 40.8% of all intracranial tumors, with 18.3% classified as WHO Grade II (1).

Diagnostic criteria for WHO grade II meningiomas have been revised in 2007, 2016, and 2021 and, as it stands now, include: 4–19 mitoses per 10 high-power fields, evidence of brain invasion, or at least three atypical features (increased cellularity, small cells with a high nuclear-to-cytoplasmic ratio, prominent nucleoli, patternless or sheet-like growth, foci of spontaneous or geographic necrosis). Clear cell and chondroid are also classified as grade II (2–5). Hence, long-term retrospective series have the shortcoming of applying the grading criteria at the time of surgery. WHO grade II meningiomas are biologically heterogeneous and demonstrate unpredictable clinical behavior, with recurrence rates significantly higher than WHO grade I (3). Maximal surgical resection remains the therapeutic cornerstone, yet the value of adjuvant radiotherapy after surgery has been the subject of debate and contradictory reports (6–10). Current guidelines from the European Association of Neuro−Oncology (EANO) and the National Comprehensive Cancer Network (NCCN) endorse either observation or irradiation after gross−total resection (GTR) and recommend radiation after subtotal resection (STR) (11, 12).

However, no universal consensus exists, and the supporting evidence is mainly retrospective and inconsistent. Even reports from the same institution reached directly opposing recommendations; one study reported decreased local recurrence in patients undergoing GTR and no benefit after STR (13), whereas another found the opposite, supporting the selective use of adjuvant radiation, particularly after STR (14).

Recent advances in molecular biology and genetics have significantly contributed to the prediction of meningioma behavior, leading to the recognition of methylation and the integration of genetic classification for a better definition of outcome and risk factors. However, these are available only recently, so they cannot be incorporated in long-term follow-up studies yet.

The present study addresses the long-term outcomes by examining a 35-year cohort of WHO grade II meningiomas treated at a single institution. We evaluate the impact of adjuvant radiation on progression-free survival (PFS) and cause−specific survival (CSS) in relation to the extent of resection and proliferative activity, as measured by the Ki−67/MIB1 index.

## 2 Materials and methods

### 2.1 Patient selection, treatments, and follow-up

This is an observational study that retrospectively analyzed a cohort of patients with WHO grade II meningiomas who underwent surgical treatment between January 1990 and December 2023.

Inclusion criteria included: a confirmed histological diagnosis of WHO grade II meningioma based on the classification at the time of first surgery; surgical resection, with or without adjuvant radiation therapy; and a minimum postoperative follow-up period of 12 months. Patients with a history of prior cranial irradiation, tumors associated with genetic syndromes, or WHO grade I or III meningiomas were excluded. All patients underwent surgical resection. The first surgical procedure was considered the initial intervention for the purposes of this study, regardless of whether it was performed at our institution or at an outside hospital. The extent of resection was determined based on the surgeon’s operative reports and postoperative magnetic resonance imaging (MRI), categorized as GTR or STR. Simpson grading was not applicable due to inconsistent reporting in operative notes.

Radiation therapy was categorized as stereotactic fractionated conformal radiotherapy (SFRT), stereotactic radiosurgery (SRS), or fractionated proton beam therapy (FPB). Radiation doses were recorded and expressed in Gray (Gy) and Gray Equivalent (CGE). Postoperative MRI was typically performed within 48 hours of surgery, followed by imaging at 3, 6, and 12 months, and then annually or biannually, or additionally as clinically indicated. Tumor recurrence was defined radiographically by progression on follow-up MRIs.

Clinical control was defined as the absence of further therapeutic interventions following the initial treatment, such as surgery alone or surgery plus adjuvant radiation. The concept of “clinical control”, usually reported as “control”, is applied in neuro-oncology for meningioma treatment (15–18).

### 2.2 Endpoints and statistical analysis

The statistical analyses were conducted using Prism 10 (GraphPad Software, Inc.), and semi-parametric models of the cause-specific hazard functions were obtained using the R packages mstate 14, survival 15, and coxphf 16. Descriptive statistics, including t-tests (Welch’s correction when appropriate) and Fisher’s exact test, were used to compare baseline characteristics between two treatment groups, stratified by the administration of adjuvant radiation therapy. Primary time-to-event endpoints were PFS, defined as time from surgery to radiological progression, and CSS, defined as time from surgery to death from meningioma. Mortality was confirmed through records and the Social Security Death Index. This approach ensured that survival estimates were not biased by unrelated causes of death.

Survival analyses were conducted using the Kaplan–Meier method, with differences between treatment groups assessed using the log-rank test. Subgroup analyses were conducted based on the extent of resection (GTR vs. STR) and Ki-67 proliferation index (<10% vs. ≥10%). A further subgroup analysis was performed comparing outcomes between irradiated patients who underwent GTR and those who underwent STR. Univariable and multivariable Cox proportional hazards models were applied to identify independent predictors of PFS and OS. The p-value threshold of<0.05 also appears to apply to the log-rank, univariable, and multivariable Cox analysis. In the multivariable analysis, the variables were age, sex, size, and tumor location (convexity, skull base, or falx/parasagittal).

Furthermore, the data were fit to competing risk models by estimating cause-specific hazard functions (19, 20). These are generalizations, allowing for transitions between multiple states, of the well-known hazard function applied in time-to-event analysis, from an initial state to a terminal state. The initial state for Model 1 was surgery, and the terminal states were 1) progression, 2) death caused by meningioma, and 3) death by other causes. This model allows for the evaluation of the effect of the covariates on the surgery-to-progression hazard function while accounting for the competing risk of death by meningioma or other cause. The initial state for Model 2 was surgery, and the terminal states were 1) death caused by meningioma, and 2) death by other causes. It allows for an evaluation of the effect of the covariates on the hazard of death by meningioma accounting for the competing risk of death by other causes. The variables used in the regression models were age, sex, the maximum diameter of the tumor at diagnosis, surgery type (GTR or STR), type of adjuvant radiation (None, FPB, SFRT, SRS), and KI67%MIB-1 index, which was converted to a binary variable with a threshold of 10%. The continuous variables, age and maximum diameter of the tumor, were centered at their mean values. The reference values of the factor variables were sex-male, surgery-STR, Radiation_type. None, and KI67 below 10%. Radiation types 1, 2, and 3 in the tables are FPB, SFRT, and SRS, respectively.

The Cox Proportional Hazards (PH) model is often used for time-varying analysis. The hazard function at time t is the probability density per unit time of transitioning from a given state to another state conditioned on being in the given state at time t. The simplest version of the Cox PH with covariates $\left(x_{1},\cdots ,x_{n}\right)$ assumes that the effects of time are captured in the baseline hazard function, λ_0_(*t*), and it is written as


$$\lambda \left(t\right)=\lambda_{0}\left(t\right)e^{\sum_{i=1}^{n}\alpha_{i}x_{i}}.$$

The hazard ratios (HR) are $e^{\alpha_{i}}$. If $e^{\alpha_{i}}>1$, then covariate *x_i_* is associated with increased hazard, and if $e^{\alpha_{i}}<\;1$, then covariate *x_i_* is associated with diminished hazard.

## 3 Results

### 3.1 Demographics and clinical features

The cohort consisted of 318 WHO grade II meningiomas in 318 patients (none of whom harbored more than one meningioma), of whom 104 (33%) received adjuvant radiation therapy. The median age was 68.5 years (interquartile range [IQR], 58-77), and 62% of the patients were female. The median maximum diameter was 4 cm (IQR, 3-5.5 cm). All patients underwent surgical resection, with GTR achieved in 193 cases (61%). Among the 267 tumors with available Ki-67 proliferation indices, the median value was 9% (IQR, 5%–15%). Of the 193 patients who underwent GTR, 39 (20%) received adjuvant radiation, whereas 65 of the 125 patients with STR (52%) were treated with adjuvant radiation. A total of 18 patients experienced malignant progression to WHO grade III, of whom 17 had been treated with adjuvant radiotherapy; 4 had undergone GTR and 13 STR.

Demographics and Clinical Features are reported in **Table 1**.

**Table 1:** Demographics and clinical features of 318 WHO grade II meningiomas.

Variable	Total	Surgery	Surgery + adjuvant radiation	p-value
pts n (%)	318	214 (67%)	104 (33%)	
Age (y) Median (IQR)	68.5 (58-77)	69 (57-79)	68 (58-76.5)	0.44
Female, n (%)	198 (62%)	137 (64%)	61 (59%)	0.38
Size (cm), Median (IQR)	4 (3-5.5)	3.9 (2.7-5.3)	4.5 (3.4-5.8)	0.09
Location, n (%)				
Convexity	151 (47%)	102 (48%)	49 (47%)	0.16
Skull base	94 (30%)	67 (31%)	27 (26%)	
Falcine/parasagittal	68 (21%)	40 (19%)	28 (27%)	
Intraventricular	5 (2%)	5 (2%)	/	
Ki67 (%) *, Median (IQR)	9 (5-15)	8 (5-13)	12.7 (7.4-20)	0.0004*
Extent of resection, n (%)				
GTR	193 (61%)	154 (72%)	39 (38%)	<0.0001*
STR	125 (39%)	60 (28%)	65 (62%)	
Malignant Progression WHO II-WHO III	18 (6%)	1 (0.5%)	17 (16%)	<0.0001*

*Data are missing for 26 cases that did not receive radiation and 25 cases that received adjuvant radiation. 51 (16%) in total.

The two groups differed significantly in terms of Ki-67 proliferation index, extent of resection, and malignant transformation rate.

GTR, Gross Total Resection; IQR, Interquartile Range; n, number; Pts, patients; STR, Sub Total Resection; y, years.

Among the group that underwent surgery and adjuvant radiation, 83 patients (80%) were treated with SFRT, 7 (8%) with SRS, and 13 (12%) with FPB. One patient received brachytherapy as the primary adjuvant modality. The most commonly administered dose was 59.4 Gy (26%), with a median total dose of 59.4 Gy (IQR, 54–60 Gy). Radiation therapy was completed at a median of 3.0 months following surgery (IQR, 2–5 months).

### 3.2 Recurrence rates

At a median follow-up of 5.1 years (mean, 6.3 years; IQR, 2.2–8.8 years), clinical control was achieved in 197 patients (92%) treated with surgery alone, including 145 who underwent GTR. In contrast, in the group that received adjuvant radiation, clinical control was observed in 43 cases (41%), of whom 24 had undergone STR.

Of the 193 patients who achieved GTR, 48 (25%) experienced tumor recurrence, with a median time to recurrence of 2.2 years (IQR, 1.0–5.4); this included 20 recurrences in the surgery-only group and 28 in the adjuvant radiation group.

Among patients who underwent STR, 62 (50%) experienced recurrence, occurring at a median of 2.6 years postoperatively (IQR, 1.2–5.2); 15 recurrences in the surgery-only group and 47 in those treated with both surgery and adjuvant radiation.

### 3.3 Progression-free survival analysis

Regarding the survival analysis, in the cohort treated with surgery alone, the actuarial PFS rates were 61% at 3 years, 45% at 5 years, and 14% at 10 years. In comparison, patients who received adjuvant radiotherapy following surgery demonstrated lower PFS rates, with 50% at 3 years, 33% at 5 years, and 9% at 10 years.

**Figure 1:**
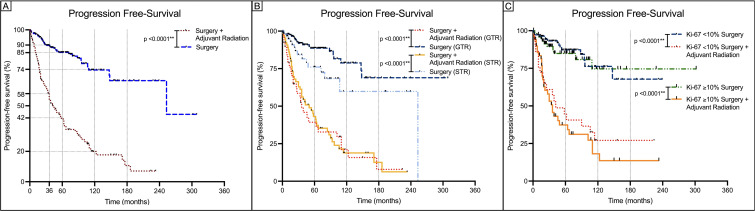
Kaplan-Meier curves of progression-free survival **(A)** and subanalyses based on the extent of resection **(B)** and Ki-67 proliferative index **(C)**. GTR, gross total resection; STR, subtotal resection.

The Kaplan–Meier analysis confirmed that adjuvant radiotherapy was significantly associated with a higher recurrence rate, as demonstrated by the log-rank test (p< 0.0001). The Kaplan–Meier estimated PFS rates for the surgery-alone group were 90%, 85%, and 74% at 3, 5, and 10 years, respectively. In contrast, the surgery plus adjuvant radiotherapy group demonstrated lower PFS rates of 58%, 42%, and 20% at 3, 5, and 10 years, respectively (**Figure 1A**).

The subanalysis stratified by extent of resection proved that adjuvant radiotherapy was significantly related to reduced PFS following both GTR (log rank p< 0.0001) and STR (log rank p< 0.0001). (**Figure 1B**) Among patients who received adjuvant radiation, there was no statistical difference in terms of PFS between those who underwent GTR and those who underwent STR (p = 0.833).

A similar association was observed in patients with Ki-67<10% and ≥10%, where adjuvant radiation was likewise associated with increased recurrence rate: log-rank, p< 0.0001 (**Figure 1C**).

**Supplementary Table 1** summarizes the associations between adjuvant radiotherapy and PFS in both univariable and multivariable analyses. Univariable proportional hazards regression analyses reported a strong statistical association between adjuvant radiotherapy and PFS (hazard ratio [HR], 5.42; 95% CI, 3.64–8.23; p< 0.0001). This association remained robust in multivariable analysis after adjusting for age, sex, tumor size, and location (HR, 5.20; 95% CI, 3.41–8.14; p< 0.0001).

Each radiation modality was independently associated with higher recurrence risk in both univariable and multivariable COX proportional regression analyses: SFRT - univariable HR, 5.04; 95% CI, 3.32–7.78; p< 0.0001; multivariable HR, 4.96; 95% CI, 3.19–7.86; p< 0.0001; SRS - univariable HR, 9.10; 95% CI, 3.67–19.54; p< 0.0001; multivariable – HR, 7.74; 95% CI, 2.98–17.74; p< 0.0001; FPB - univariable HR, 6.12; 95% CI, 2.86–11.98; p< 0.0001; multivariable HR, 5.49; 95% CI, 2.44–11.48; p< 0.0001. Regarding the extent of resection, adjuvant radiotherapy was significantly associated with worse PFS following GTR (HR, 6.91; 95% CI, 3.90–12.47; p< 0.0001) and STR (HR, 3.39; 95% CI, 1.91–6.40; p< 0.0001). These remained significant in the multivariable analysis after adjusting for age, sex, tumor size, and location (GTR: HR, 6.02; 95% CI, 3.26–11.38; p< 0.0001; STR: HR, 3.12; 95% CI, 1.65–6.48; p = 0.0011).

Patients with a Ki-67 index ≥10% exhibited a substantially higher risk of recurrence after adjuvant radiation (univariable HR 5.86; 95% CI, 3.05–12.18; p< 0.0001, and multivariable HR 5.74; 95% CI, 2.79–12.87; p< 0.0001). Also, patients with Ki-67<10% demonstrated an increased recurrence rate after adjuvant radiation (univariable - HR 5.29; 95% CI, 2.68–10.66; p< 0.0001; and multivariable analysis HR, 5.67; 95% CI, 2.65–12.48; p< 0.0001. Adjuvant radiotherapy was a significant risk factor for recurrence following GTR in patients with a Ki-67 index ≥10%, as shown in both univariable (HR 8.26; 95% CI, 3.42–22.93; p< 0.0001) and multivariable analyses (HR 5.99; 95% CI, 2.20–19.39; p = 0.0010). Similarly, in patients with Ki-67<10% who underwent GTR, adjuvant radiation was associated with significantly worse PFS in both univariable (HR 4.77; 95% CI, 1.47–13.46; p = 0.0046) and multivariable models (HR 5.11; 95% CI, 1.47–16.10; p = 0.0062).

Following STR, adjuvant radiotherapy was not significantly associated with PFS in patients with Ki-67 ≥10% (HR 2.73; 95% CI, 1.05–8.46; p = 0.053). For patients with Ki-67<10% (n = 50), adjuvant RT was a significant risk factor for recurrence in univariable analysis (HR 5.13; 95% CI, 1.82–18.18; p = 0.0041), but this association did not remain significant in the multivariable model (HR 2.91; 95% CI, 0.93–11.33; p = 0.085). In contrast, in patients with Ki-67 ≥10% who underwent STR, postoperative RT, which was not significant in the univariable analysis, became significant in the multivariable model as a risk factor for reduced PFS (HR 0.23; 95% CI,??–0.63; p = 0.0059).

Following recurrence, not all patients required immediate intervention. Of the total cases with radiographic progression, 30 were initially observed. Salvage treatments included radiation alone in 22 patients, surgery alone in 32 patients, and combined surgery with salvage radiation therapy in 26 patients.

### 3.4 Cause-specific survival analysis

Regarding actuarial overall survival (OS), in the surgery-alone group, the 5-, 10-, and 15-year OS actuarial rates were 54%, 20%, and 8%, respectively. In the surgery plus adjuvant radiation group, OS was 66% at 5 years, 32% at 10 years, and 18% at 15 years. Although the actuarial OS data suggested a modest long-term survival benefit with adjuvant radiotherapy, the actuarial cause-specific survival (CSS) analysis demonstrated worse outcomes in the irradiated cohort. Specifically, the actuarial CSS rates for the surgery-alone group were 76%, 40%, and 29% at 5, 10, and 15 years, respectively, whereas the surgery plus adjuvant radiotherapy group demonstrated lower CSS rates of 72%, 37%, and 18% at 5, 10, and 15 years, respectively, highlighting a survival difference in favor of the surgery-alone group.

**Figure 2:**
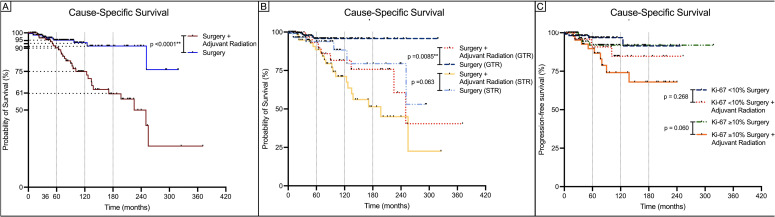
Kaplan-Meier curves of cause-specific survival **(A)** and subanalyses based on the extent of resection **(B)** and Ki-67 proliferative index **(C)**. GTR, gross total resection; STR, subtotal resection.

This negative association between adjuvant radiotherapy and survival was further confirmed by the Kaplan–Meier log-rank analysis (p< 0.0001);. The Kaplan–Meier estimated CSS rates for the surgery-alone group were 95%, 93%, and 91% at 5, 10, and 15 years, respectively, compared with 90%, 75%, and 61% for the surgery plus adjuvant radiotherapy group at the same time points. (**Figure 2A**).

The subanalysis based on extent of resection, comparing patients who underwent GTR with or without adjuvant radiation therapy, demonstrated that adjuvant radiation was associated with worse CSS (log-rank, p = 0.0085). In contrast, among patients who underwent STR, no significant difference in CSS was observed between those who received adjuvant radiation and those who did not (log-rank, p = 0.063) (**Figure 2B**).

Within the cohort of patients who received adjuvant radiotherapy, no statistically significant difference in CSS was observed between those who underwent GTR and those who underwent STR (p = 0.176).

In the survival analysis, the log-rank test didn’t show a significant difference in CSS when patients were stratified by Ki-67 index ≥10% versus<10% (log-rank p = 0.268, p = 0.060). (**Figure 2C**).

**Supplementary Table 2** presents the relationships between adjuvant radiotherapy and CSS in both univariable and multivariable analyses. In the Cox proportional hazards regression analyses, the association between adjuvant radiotherapy and CSS remains statistically significant in both the univariable and multivariable models, after adjustment for covariates (HR 4.02, 95% CI 2.05-8.47, p< 0.0001; HR 3.99, 95% CI 1.94-8.91, p = 0.0003). Regarding the radiation modality, SFRT and SRS were associated with a high risk of death in both univariable (SFRT: HR, 4.32; 95% CI 2.16-9.22; p< 0.0001; SRS: HR, 5.07; 95% CI 1.13-16.57; p = 0.0139) and multivariable (SFRT: HR, 4.18; 95% CI 2.01-9.38; p = 0.0002; SRS: HR, 4.22; 95% CI 0.89-15.01; p =0.0387) analyses, while the FPB was not identified as a risk factor. After GTR radiation was found to be a risk factor for mortality (HR 4.61; 95% CI, 1.50-15.50; p = 0.0085), this remained significant on multivariable testing (HR, 3.97; 95% CI 1.21-14.03; p = 0.0237). Adjuvant radiation did not influence survival after STR in the univariable analysis (HR, 2.38; 95% CI, 1.01-6.55; p = 0.0634). In contrast with the Kaplan-Meier analysis, in the subgroup Cox univariable analysis, adjuvant radiotherapy did not reach statistical significance as a risk factor for decreased survival in either subgroup Ki-67 ≥10% (HR, 3.09; 95% CI, 1.00-11.43; p = 0.060) and< 10% (HR, 2.35; 95% CI, 0.45-10.87; p=0.268). Adjuvant radiotherapy was not associated with an increased risk of death in patients with Ki-67<10% meningiomas, either after GTR (HR 1.19e-11; 95% CI, not available to 16.15; p > 0.9999) or STR (HR 1.59; 95% CI, 0.25–12.26; p = 0.616).

Similarly, no significant association was observed in the Ki-67 ≥10% group, either following GTR (HR 2.26; 95% CI, 0.57–13.36; p = 0.2060) or STR (HR 2.87; 95% CI, 0.44–55.69; p = 0.3395).

### 3.5 Competing risk models

Hazard ratios, 95% confidence intervals, and p-values of the coefficients for each of the hazard functions for Models 1 and 2 are reported in **Table 2** and **3**, respectively. The prefix.n on the variable names indicates the hazard function; n = 1,2,3 denotes the hazard functions corresponding to the transitions from surgery to progression, death by meningioma without having a progression, and death by other causes without a progression, respectively. For Model 2, n = 1 or 2 denotes the transitions from surgery to death by meningioma or death by other cause, respectively. Using a p-value threshold of 0.05, one sees from **Table 2**, that the hazard of progression increases for females, greater tumor size, and all of the adjuvant radiation types (**Table 2**).

**Table 2:** Summary of model 1 regression coefficients.

Term	log-HR	HR	HR 95% CI	p-value
Age_y:c.1	0.002	1.002	0.988-1.017	0.757
Age_y:c.2	0.061	1.063	1.011-1.103	0.019
Age_y:c.3	0.042	1.043	1.017-1.068	<0.001*
Sex_F_M_.1	0.599	1.820	1.223-2.727	0.003*
Sex_F_M_.2	-1.327	0.265	0.051-0.882	0.028*
Sex_F_M_.3	-0.579	0.561	0.284-1.060	0.075
MaxDiameter_cm:c.1	0.142	1.153	1.028-1.286	0.015*
MaxDiameter_cm:c.2	0.248	1.282	0.779-1.794	0.294
MaxDiameter_cm:c.3	0.004	1.004	0.801-1.238	0.974
GTR.1	-0.288	0.750	0.498-1.121	0.161
GTR.2	-1.091	0.336	0.063-1.175	0.093
GTR.3	0.299	1.349	0.704-2.571	0.366
RT_type1 (FPB).1	1.645	5.181	2.203-10.964	<0.001*
RT_type2 (SFRT).1	1.489	4.433	2.854-6.914	<0.001*
RT_type3 (SRS).1	2.128	8.398	4.568-14.946	<0.001*
KI67B.1	0.293	1.340	0.895-2.011	0.155
KI67B.2	-1.990	0.137	0.013-0.690	0.013
KI67B.3	-0.203	0.816	0.409-1.579	0.552

CI, 95% Confidence Intervals; F, Female; FPB, Fractionated Proton Beam; GTR, Gross Total Resection; HR, Hazard Ratios; log-HR; log-hazard ratios; M, Male; RT, Radiotherapy; SFRT, Stereotactic Fractionated Radiotherapy; SRS, Stereotactic Radiosurgery; y, years-old. *statistically significant.

**Table 3:** Summary of model 2 regression coefficients.

Term	log-HR	HR	HR 95% CI	p-value
Age_y:c.1	0.002	1.002	0.988-1.017	0.761
Age_y:c.2	0.087	1.091	1.022-1.168	0.008
Sex_F_M_.1	0.604	1.829	1.255-2.663	0.002*
Sex_F_M_.2	-1.340	0.262	0.046-0.956	0.042
MaxDiameter_cm:c.1	0.143	1.154	1.030-1.285	0.014*
MaxDiameter_cm:c.2	0.433	1.542	0.853-2.580	0.144
GTR.1	-0.290	0.748	0.501-1.108	0.148
GTR.2	-1.277	0.279	0.045-1.070	0.064
RT_type1 (FPB).1	1.645	5.181	2.235-10.710	<0.001*
RT_type2 (SFRT).1	1.490	4.437	2.919-6.720	<0.001*
RT_type2 (SFRT).2	-1.074	0.342	0.003-2.541	0.372
RT_type3 (SRS).1	2.133	8.440	4.651-14.728	<0.001*
RT_type3 (SRS).2	-1.763	0.172	0.001-2.727	0.257
KI67B.1	0.296	1.344	0.902-1.998	0.146
KI67B.2	-2.361	0.094	0.005-0.864	0.035

CI, 95% Confidence Intervals; F, Female; FPB, Fractionated Proton Beam; GTR, Gross Total Resection; HR, Hazard Ratios; log-HR; log-hazard ratios; M, Male; RT, Radiotherapy; SFRT, Stereotactic Fractionated Radiotherapy; SRS, Stereotactic Radiosurgery; y, years-old. *statistically significant.

From **Table 3**, one sees an increasing hazard of death by meningioma for females, larger tumor size, and any of the types of adjuvant radiation. One also sees an increasing hazard of death by other causes with increasing age and a decreasing hazard of death by other causes for females, and if the KI67%MIB-1 index exceeds 10%. FBP was excluded from hazard 2 for computational reasons (**Table 3**).

## 4 Discussion

### 4.1 Adjuvant radiation effectiveness on progression-free survival and overall survival in grade II

The practice of adjuvant radiation therapy for WHO grade II meningioma has common but debatable effectiveness in preventing recurrence. A key focus in radiotherapy for WHO grade II meningiomas has been its role and effectiveness after GTR or STR. Recent guidelines seem to recommend adjuvant radiation after subtotal and optional radiation after total removal (11, 12). This paradigm of not administering adjuvant radiation after GTR has been the recommendation of several authors (14, 21).

On the contrary, other authors demonstrated radiation benefit only after GTR and questioned the effect on PFS after STR (13). Conversely, other studies favor upfront adjuvant radiotherapy, irrespective of the extent of resection. In 2017, Bagshaw et al. reported improved local control with the use of adjuvant RT, independent of Simpson grade, emphasizing the difficulty of managing recurrences once they occur; thus, they concluded that an initial aggressive therapeutic approach may be warranted to optimize long-term disease control (9).

More recently, meta-analysis by Song et al. in 2021, encompassing 3,078 patients across 2,307 studies, demonstrated that postoperative RT significantly improved both PFS (p = 0.01) and OS (p = 0.007) after GTR, as well as after STR (PFS p< 0.00001; OS p = 0.01), supporting its benefit regardless of resection extent with 55 average months of follow-up (22). This contrasts with several authors who have emphasized that its benefit is largely confined to cases of STR (14, 23). On the other hand, in our analysis, adjuvant radiation was significantly associated with reduced PFS, regardless of the extent of resection. Our findings corroborate other reports, like Graffeo et al. in 2017, who studied 69 patients and found that clinical surveillance after GTR did not increase the risk of recurrence or mortality, suggesting that sole observation may be a safe alternative to adjuvant radiation (24). Supporting this, Poulen et al. (2020) reported significantly longer PFS in the non-irradiated group compared to those receiving adjuvant radiation (60.4 vs. 39.1 months, P = 0.02) (25).

Our competing-risk model demonstrated that all radiation modalities were associated with an increased risk of transition from surgery to progression. Most of the studies report on the OS, however, it will be more relevant to report the CSS, which, unfortunately, fewer articles detail (13, 26–28).

In our study, a marked effect on CSS was observed in patients receiving adjuvant radiation, both in the overall cohort and in the GTR subgroup. In contrast, adjuvant radiation had no significant impact on survival after STR. These findings support the use of clinical surveillance as a preferred postoperative strategy, particularly after GTR.

In 2019, Reddy et al. conducted an extensive review based on SEER data from 1973 to 2015 and found that adjuvant radiotherapy did not significantly improve survival in atypical meningiomas, neither after GTR nor STR (29). Another analysis in 2024 of the SEER database by Quin et al. evaluated patients with solitary intracranial atypical meningiomas treated with GTR or STR, with or without postoperative radiotherapy. In patients<60 years, postoperative radiotherapy was associated with improved OS following STR, with survival comparable to GTR alone. In contrast, in patients ≥60 years, postoperative radiotherapy was identified as an independent risk factor for OS after both GTR and STR (30). In the same year, Li et al. analyzed SEER data to assess the impact of adjuvant proton beam radiotherapy on OS in patients with primary solitary intracranial atypical meningioma, with a focus on age. Adjuvant proton beam radiotherapy was associated with reduced mortality in patients ≤55 years, but increased mortality in those >55 years. Multivariable Cox analysis confirmed adjuvant beam radiotherapy as a protective factor in ≤55 patients and a risk factor in >55 patients (31).

Champeaux et al., consistent with our findings, also observed no survival benefit from radiotherapy, including after incomplete resection (10). Supporting this, Poulen et al. (2020) reported no OS difference between irradiated and non-irradiated patients (P = 0.93) (25).

Furthermore, in our competing-risk model, all types of radiation were associated with a higher risk of transition from surgery to meningioma-specific death.

### 4.2 Extent of resection on the role of radiation

The prognostic significance of the extent of resection in meningioma surgery is established through the literature (6, 7, 32). The importance of radical tumor removal in WHO grade II was reinforced by Aizer et al., who reported superior 5-year survival rates in patients with atypical meningiomas undergoing GTR compared to those receiving STR (91.3% vs. 78.2%), with GTR independently associated with reduced all-cause mortality in multivariable analysis (8). Regarding WHO grade II meningiomas, the literature suggests that the extent of resection is a pivotal factor in outcome and, consequently, in the recommendation. While some articles clearly advocate irradiation after STR, others see its benefit only if GTR was obtained. However, since the term GTR does not imply complete tumor removal in meningioma, it would be categorized as Simpson grade III, leaving the tumor in the bone and the adjacent dura. Hence, it is factually STR since it leaves residual. Accordingly, it is not surprising that there are contradictory conclusions in this regard in the literature.

On the other hand, a true total removal (Simpson grade I meningioma) might indeed render radiation therapy unneeded. Unfortunately, our study, similar to many others, was not able to categorize the cases strictly on Simpson grading. Song et al, reported a subgroup analysis showing no change in PFS in patients treated with adjuvant radiation who underwent Simpson grade I and II resection (p=0.22) but significant improvement in patients undergoing Simpson grade III resection, which otherwise would have been called GTR (p=0.02) (22). Also, in the study by Gupta et al., among 298 patients with GTR, adjuvant radiation did not seem to confer an improvement in progression, but when stratified by Simpson grade, there may have been a statistically insignificant trend toward longer PFS after adjuvant radiation in cases with Simpson grade III resection (14).

The question here is whether this could be explained by the fact that in Simpson grade III, the remaining tumor is less in volume and even invisible on the imaging, and in this situation, radiation might be effective in halting the growth.

### 4.3 Molecular biology

The rapid evolution of molecular characterization of meningiomas, through the identification of genetic alterations including NF2, CDKN2A/B deletions, TERT promoter mutations, as well as DNA methylation profiling, will offer superior prognostic value. It should pre-identify the more aggressive WHO grade II meningiomas and possible resistance or response to radiotherapy. Currently, reclassification is underway, integrating histopathology with molecular markers and genetic features to enhance prognostic accuracy and refine risk stratification, ultimately guiding more individualized treatment strategies (33–39).

However, retrospective studies can still offer value until sufficient data and follow-up length are available to analyze under the new paradigm.

In our study, we incorporated Ki-67/MIB-1 index analysis as a variable, which serves as a marker of cellular proliferative activity. Elevated Ki-67 levels are directly associated with aggressive tumor behavior, with values >10% predicting locoregional progression following SRS, suggesting a possible role in treatment stratification and SRS dose modulation (40–42). Gupta et al. reported improved PFS with adjuvant RT in patients with Ki-67 indices >10%, supporting its selective use in high-risk subgroups (14). Similarly, Wang et al. evaluated recurrence rates based on the MIB-1 index, using a threshold of 8%. Among patients who received adjuvant RT, recurrence occurred in 50% of those with MIB-1 >8%, compared to 20% with MIB-1<8%. Conversely, among patients who did not receive adjuvant radiation, recurrence rates were 100% for MIB-1 >8% and 50% for MIB-1<8% (39). These findings suggest that patients with elevated proliferative indices may derive greater benefit from adjuvant radiation.

In our analysis, adjuvant radiotherapy was associated with increased recurrence risk, independent of Ki-67 expression (≥10% or<10%) and extent of resection (GTR or STR). Notably, Ki-67 did not significantly impact CSS.

### 4.4 Future perspectives

Despite growing interest, high-quality evidence regarding the optimal use of adjuvant radiotherapy in meningiomas remains limited. Greater clarity is expected from two prospective, randomized phase III trials: the recently completed ROAM/EORTC 1308 trial and the ongoing NRG-BN003 trial, projected for completion in June 2027. Unfortunately, these studies do not use Simpson grading to assess the extent of resection. To date, the only completed multicenter prospective trial was conducted by Rogers and colleagues. This study represents the first clinical outcomes report from this cooperative group, showing that patients with intermediate-risk meningioma treated with postoperative irradiation achieved excellent 3-year PFS. The results support the role of postoperative RT in both newly diagnosed, GTR resected WHO grade II meningiomas and recurrent WHO grade I meningiomas, regardless of resection extent (NCT00895622). Unfortunately, it is not built on Simpson grading resection and has a relatively short follow-up period (43).

In the absence of level 1 evidence, the decision to administer adjuvant RT should be individualized, balancing potential oncologic benefit against treatment-related morbidity, and guided by resection extent, tumor biology, and patient-specific factors.

### 4.5 Study limitations

This study is subject to the inherent limitations of a retrospective study that spans over many years, including potential selection and information biases. The decision to administer adjuvant RT was not randomized, raising the possibility that patients with more aggressive disease were preferentially selected for radiation. Furthermore, the evolution of the WHO classification system for meningiomas over the study period represents an important limitation. Diagnostic criteria for WHO grade 2 meningiomas have changed over time, and consequently, some tumors classified as grade 2 in earlier decades may not meet current WHO criteria. This temporal heterogeneity may have introduced classification bias and should be considered when interpreting long-term outcomes. Additional confounders include variability in surgical expertise, lack of standardized use of the Simpson grading system to quantify the extent of resection, and heterogeneous radiation techniques applied over the extended study period. The absence of comprehensive molecular profiling, particularly in earlier years, further limits our ability to assess the prognostic impact of genetic alterations.

## 5 Conclusion

In this observational study, adjuvant radiotherapy for WHO grade II meningiomas was significantly associated with decreased PFS and CSS. In particular, the extent of resection remains the most important independent prognostic factor and therefore represents a key element in guiding treatment strategy and long-term management.

These findings challenge some current treatment paradigms and highlight the urgent need for personalized approaches and robust prospective trials.

## Data Availability

The raw data supporting the conclusions of this article will be made available by the authors, without undue reservation.
