# Supplementary material for: Adjuvant radiotherapy for WHO grade II meningiomas: the unanswered question
Source: Front Oncol. 2026 Jun 24;16:1815992. doi: 10.3389/fonc.2026.1815992 (PMC13341486; doi:10.3389/fonc.2026.1815992)
Supplement: Supplementary file 1 [file Table1.docx]

**Table S1.** Univariable and Multivariable Cox Proportional Hazards Regression Analyses of the Association Between Adjuvant Radiotherapy and Progression-Free Survival

| **Value** | **Univariate** | | | **Multivariate** | | |
| --- | --- | --- | --- | --- | --- | --- |
|  | **HR** | **95% CI** | **p-value** | **HR** | **95% CI** | **p-value** |
| Adjuvant RT | 5.42 | 3.64-8.23 | **p < 0.0001**** | 5.20 | 3.41–8.14 | **p < 0.0001**** |
| GTR | 6.91 | 3.90–12.47 | **p < 0.0001**** | 6.02 | 3.26–11.38 | **p < 0.0001**** |
| STR | 3.39 | 1.91–6.40 | **p < 0.0001**** | 3.12 | 1.65–6.48 | **p = 0.0011*** |
| SFRT | 5.04 | 3.32–7.78 | **p < 0.0001**** | 4.96 | 3.19–7.86 | **p < 0.0001**** |
| SRS | 9.10 | 3.67–19.54 | **p < 0.0001**** | 7.74 | 2.98–17.74 | **p < 0.0001**** |
| FPB | 6.12 | 2.86–11.98 | **p < 0.0001**** | 5.49 | 2.44–11.48 | **p < 0.0001**** |
| Ki-67 ≥10% | 5.86 | 3.05–12.18 | **p < 0.0001**** | 5.74 | 2.79–12.87 | **p < 0.0001**** |
| GTR | 8.26 | 3.42–22.93 | **p < 0.0001**** | 5.99 | 2.20–19.39 | **p = 0.0010**** |
| STR | 2.73 | 1.05–8.46 | p = 0.053 | 0.23 | ??-0.63 | **p = 0.0059*** |
| Ki-67 <10% | 5.29 | 2.68–10.66 | **p < 0.0001**** | 5.67 | 2.65–12.48 | **p < 0.0001**** |
| GTR | 4.77 | 1.47–13.46 | **p = 0.0046**** | 5.11 | 1.47–16.10 | **p = 0.0062**** |
| STR | 5.13 | 1.82–18.18 | **p = 0.0041**** | 2.91 | 0.93–11.33 | p = 0.085 |

CI: Confidence Interval; FPB: fractionated proton beam therapy; GTR: Gross Total Resection; HR: Hazard Ratio; RT: Radiation; SFRT: Fractionated Stereotactic Conformal Radiotherapy; SRS: Stereotactic Radiosurgery; STR: Sub Total Resection.
