# Supplementary material for: Adjuvant radiotherapy for WHO grade II meningiomas: the unanswered question
Source: Front Oncol. 2026 Jun 24;16:1815992. doi: 10.3389/fonc.2026.1815992 (PMC13341486; doi:10.3389/fonc.2026.1815992)
Supplement: Supplementary file 2 [file Table2.docx]

**Table S2.** Univariable and Multivariable Cox Proportional Hazards Regression Analyses of the Association Between Adjuvant Radiotherapy and Cause-Specific Survival

| **Value** | **Univariate** | | | **Multivariate** | | |
| --- | --- | --- | --- | --- | --- | --- |
|  | **HR** | **95% CI** | **p-value** | **HR** | **95% CI** | **p-value** |
| Adjuvant RT | 4.02 | 2.05-8.47 | **p < 0.0001**** | 3.99 | 1.94-8.91 | **p = 0.0003**** |
| GTR | 4.61 | 1.50-15.50 | **p = 0.0085**** | 3.97 | 1.21-14.03 | **p = 0.0237*** |
| STR | 2.38 | 1.01-6.55 | p = 0.063 | 1.94 | ??-6.63 | p = 0.25 |
| SFRT | 4.32 | 2.16-9.22 | **p < 0.0001**** | 4.18 | 2.01-9.38 | **p = 0.0002**** |
| SRS | 5.07 | 1.13-16.57 | **p = 0.0139*** | 4.22 | 0.89-15.01 | **p = 0.0387*** |
| FPB | 1.22 | 0.06-6.32 | p = 0.845 | 1.40 | 0.07-8.11 | p = 0.752 |
| Ki-67 ≥10% | 3.09 | 1.00-11.43 | p = 0.060 | 3.08 | 0.92-12.12 | p = 0.078 |
| GTR | 2.62 | 0.57- 13.36 | p = 0.206 | 1.93 | 0.30-13.76 | p = 0.48 |
| STR | 2.87 | 0.44-55.69 | p = 0.339 | 1.47 | 0.13-35.18 | p = 0.76 |
| Ki-67 <10% | 2.35 | 0.45-10.87 | p = 0.268 | 4.49 | 0.66-32.04 | p = 0.11 |
| GTR | 1.191e-011 | ??? to 16.15 | p > 0.9999 | 1.258e-013 | ??? | p > 0.9999 |
| STR | 1.590 | 0.25 to 12.26 | p = 0.616 | 7.52 | 0.38-781.2 | p = 0.24 |

CI: Confidence Interval; FPB: fractionated proton beam therapy; GTR: Gross Total Resection; HR: Hazard Ratio; RT: Radiation; SFRT: Fractionated Stereotactic Conformal Radiotherapy; SRS: Stereotactic Radiosurgery; STR: Sub Total Resection.
